# Corns on the Feet

**Published:** 1871-01

**Authors:** 


Corns on the Feet.—The following method of removing these troublesome growths is given in *La Sante:* Macerate the tender leaves of ivy in strong vinegar for eight or ten days, then apply them on the corns. This dressing should be applied twice a day, and in a few days the corns will be removed.—*Medical News.*

